# Dobutamine induced changes in aortic stiffness: influence of obesity in middle aged and elderly individuals with hypertension, diabetes or coronary artery disease

**DOI:** 10.1186/1532-429X-15-S1-O45

**Published:** 2013-01-30

**Authors:** Sujethra Vasu, Tim M Morgan, W Gregory Hundley, Kimberly Lane

**Affiliations:** 1 Cardiology, Wake Forest University School of Medicine, Winston-Salem, NC, USA; 2 Public Health, Wake Forest Unviersity School of Medicine, Winston-Salem, NC, USA

## Background

Previously, we have shown that increased visceral adiposity was an independent predictor of thoracic aortic wall thickness in elderly individuals at risk for future cardiovascular (CV) events. In this study, we sought to assess the influence of obesity on rest and stress induced changes in aortic stiffness in older individuals with hypertension, diabetes or coronary artery disease at risk for future CV events.

## Methods

We performed dobutamine CMR on 302 consecutively referred middle aged and older individuals with hypertension, diabetes, or CAD. We assessed aortic stiffness using the ratio of pulse pressure to stroke volume index (PP/SVI). Left ventricular stroke volume was assessed using cine MRI at rest and with peak stress. All analyses were performed by individuals blinded to the CMR imaging procedure and participant identifiers. The group was divided into tertiles based on BMI (< 27, 27-32, >32) among 3 age groups (55-64, 65-74, 75+). The correlation between BMI expressed in tertiles and the resting PP/SVI and the changes with dobutamine was assessed using linear regression. This was tested for an interaction with age. Values are expressed as mean±standard error; p value of <0.05 was considered significant.

## Results

**Figure 1:**
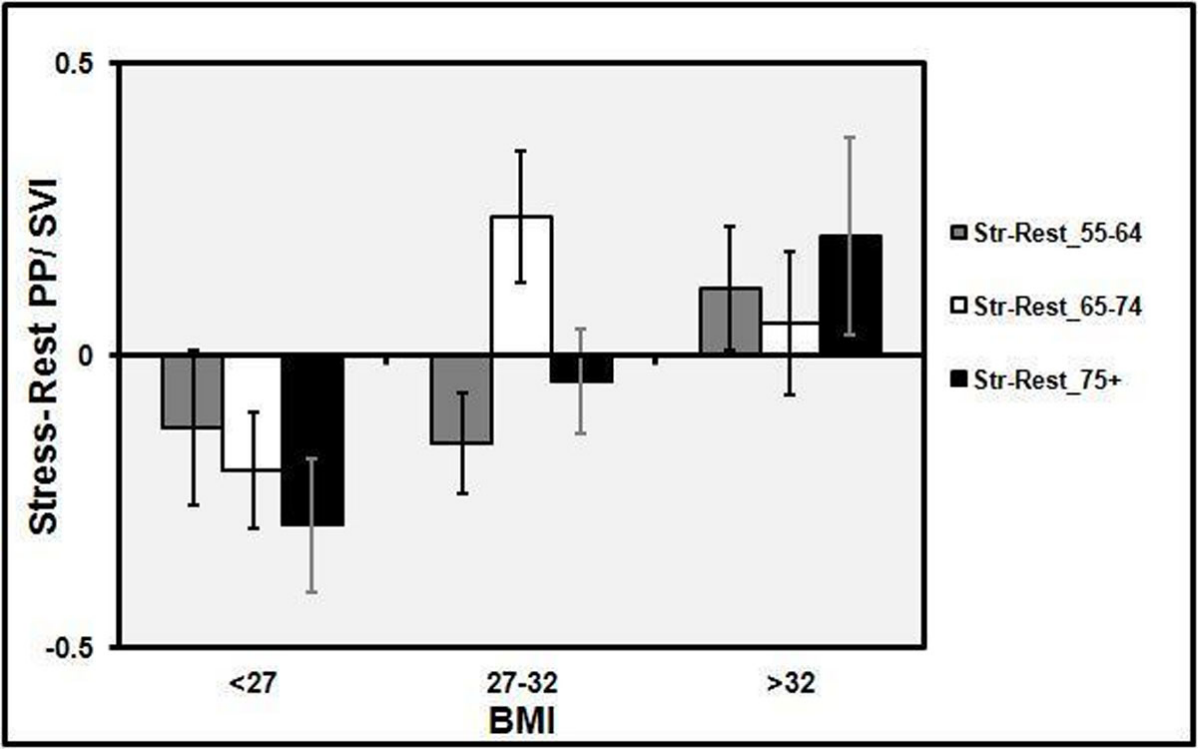
Change in PP/SVI: Stress- Rest

The demographics and results are shown in Figure 1. At rest there were no differences in the PP/SVI between the three BMI groups in all age groups. After dobutamine, subjects aged 55-64 in the highest BMI tertile (> 32) had an increase in the PP/SVI by 0.115±0.105 similar to that of subjects aged 75+ in the highest BMI tertile, 0.205±0.168 (Figure 2). In contrast, both both subjects aged 55-64 and 75+ in the low and medium BMI tertiles experienced a decrease in the PP/SVI with dobutamine, -0.122±0.133 and -0.150±0.086 for the 55-64 age group and -0.290±0.115 and -0.044±0.089 in the 75+ age group. After adjusting for age and gender, stress PP/SVI and the change in PP/SVI had a significant association with BMI (p =0.005 and p< 0.0001) respectively. Age had a significant interaction in this relationship (p = 0.02), whereas fasting glucose did not have a significant interaction.

## Conclusions

Middle aged and elderly subjects with BMI >32 have increased aortic stiffness after dobutamine stress in contrast to those with BMI <32. The prognostic significance of the increased aortic stiffness with stress in obese individuals and its relationship to ventricular remodeling deserve further study.

## Funding

NIH R01 HL076438 Dr. W Gregory Hundley.

**Table 1:** Demographics and results

Measure	BMI < 27.45 (N=98)	BMI 27.45-32 (N=101)	BMI > 32 (N=103)	p-value with BMI
Age	70.3 (8.0)	68.9 (7.8)	66.6 (8.0)	0.0003
55-64	24 (24.5 %)	35 (34.6%)	48 (46.6%)	
65-74	40 (40.8%)	38 (37.6%)	34 (33.0%)	
75+	34 (34.7%)	28 (27.7%)	21 (20.9%)	

Female	50 (51.0%)	47 (46.5%)	71 (68.9%)	0.018

Race				0.0017
Caucasian	88 (89.8%)	80 (79.2%)	71 (68.9%)	
African Am.	6 (11.1%)	17 (31.5%)	31 (30.1%)	

Prior HTN	88 (89.8%)	92 (91.0%)	98 (95.2%)	0.23

Prior DM				0.018
None	64 (65.3%)	60 (59.4%)	55 (53.4%)	
< 5 years	34 (34.7%)	33 (32.7%)	40 (38.8%)	
= 5 years	0	8 (7.9%)	8 (7.8%)	

Prior CAD	27 (27.6%)	22 (21.8%)	16 (15.5%)	0.34

SQ fat	143.1 (56.7)	202.9 (73.7)	325.5 (132.4)	< 0.0001

Visceral fat	142.6 (72.6)	197.3 (94.7)	238.4 (97.8)	< 0.0001

Taking drugs				
ACE	48 (49.0%)	50 (49.5%)	39 (37.9%)	0.27
ARB	4 (4.2%)	4 (4.2%)	3 (2.9%)	0.73
Statin	64 (65.4%)	64 (65.4%)	69 (67.0%)	0.82
Beta blocker	45 (45.9%)	45 (45.9%)	41 (39.8%)	0.43
Ca Ch. BL.	23 (23.5%)	23 (23.5%)	25 (24.5%)	0.67

				

Rest_55-64	1.618 ± 0.119	1.528 ± 0.096	1.45 ± 0.069	
Stress_55-64	1.528 ± 0.107	1.371 ± 0.103	1.513 ± 0.092	
Rest_65-74	1.719 ± 0.074	1.63 ± 0.075	1.739 ± 0.089	
Stress_65-74	1.537 ± 0.07	1.842 ± 0.102	1.701 ± 0.084	
Rest_75+	1.898 ± 0.08	1.848 ± 0.103	1.79 ± 0.088	
Stress_75+	1.694 ± 0.11	1.784 ± 0.123	1.962 ± 0.152	

